# Internet use time and subjective well-being during the COVID-19 outbreak: serial mediation of problematic internet use and self-esteem

**DOI:** 10.1186/s40359-023-01483-x

**Published:** 2023-12-11

**Authors:** Min-Pei Lin, Hsin-Yi Tseng, Yueh-Ting Lee, Wen-Ching Tang, Li-Hsuan Cheng, Jo Yung-Wei Wu, Jianing You

**Affiliations:** 1 Department of Educational Psychology and Counseling, National Taiwan Normal University, No.162, Sec. 1, Heping E. Rd., Da-an District, Taipei City, 106 Taiwan; 2 Department of Counseling and Guidance, National University of Tainan, No.33, Sec. 2, Shu- Lin St, Tainan, 700 Taiwan; 3 Good-Day Psychology Clinic, 5F., No. 167, Xialin Rd., South District, Tainan City, 702 Taiwan; 4 Center for Studies of Psychological Application, Guangdong Key Laboratory of Mental Health and Cognitive Science, Key Laboratory of Brain, Cognition and Education Sciences (South China Normal University), & School of Psychology, Ministry of Education, South China Normal University, Guangzhou, 510631 P. R. China

**Keywords:** Internet use time, Problematic internet use, Self-esteem, Subjective well-being, COVID-19 outbreak

## Abstract

The coronavirus disease (COVID-19) outbreak is a threat to adolescents’ mental health and livelihoods, and lowers their subjective well-being (SWB). Expanding on previous literatures, this study examined whether internet use time was related to SWB and whether this relationship was mediated by problematic internet use (PIU) and self-esteem during the COVID-19 outbreak. In Taiwan, the COVID-19 epidemic broke out in February, 2020. During March 2 to 27, this study recruited a total of 1,060 adolescents from junior high schools by both stratified and cluster sampling, and administered a comprehensive investigation. The results displayed that SWB was significantly and negatively predicted by internet use time. PIU fully mediated the relationship. Moreover, PIU predicted a decrease of self-esteem, which played a full mediation role between PIU and SWB. The results provide evidence in explaining how increased internet use time is associated with a greater level of PIU, which relates to lower self-esteem, correlating with lower SWB in adolescents. This study can provide reference to mental health organizations and educational agencies to design appropriate SWB promotion programs for the junior high school population in terms of epidemic prevention.

## Introduction

In attempt to mitigate the transmission of the coronavirus disease (COVID-19), caused by the severe acute respiratory syndrome coronavirus 2, many countries took drastic actions to minimize social interactions [1–5]. The repeatedly lockdown and lifting of lockdowns resulted in a sudden and unpredictable loss of many activities that created a radical change in the daily lives of adolescents such as the temporary closure of educational institutions and reorganized leisure time activities, lack of autonomy, and social isolation [6, 7]. The outbreak of COVID-19 has significantly disrupted the daily behaviors of adolescents [1, 6]. All these circumstances may lead to a decline in subjective well-being (SWB), and emerging literature that have explored SWB during the COVID-19 outbreak [8–12] indicated that the pandemic caused a negative impact on mental health [13]. Therefore, it becomes more important to highlight the transformation process of SWB during the COVID-19 outbreak for researchers to investigate mutual relationships between outcomes and antecedents of the exposure.

### The relationship between the internet use time and subjective well-being

During the COVID-19 outbreak, social distancing, school closures, and quarantine became the new worldwide norm, resulting in adolescents spending more time at home and on the Internet [14]. However, increased internet engagement may greatly affect adolescents’ SWB [15, 16]. Twenge et al. (2018) discovered that adolescents who consumed more time on electronic communication and screens (e.g., social media, the Internet, texting, gaming) and less on non-screen activities had lower SWB [15]. Furthermore, Orben and Przybylski (2019) also found a negative association between digital technology use and adolescent SWB [16]. However, Trott et al. (2022) have shown that during the COVID-19 pandemic, the relationship between screen time and an individual’s well-being might be conflicting [17]; while some agreed that increased screen time led to decreased well-being, others showed insignificant correlations. Thus, the present study examines the relationship between internet use time and adolescent SWB, and hypothesized that internet use time was negatively related to SWB.

### The mediating role of problematic internet use

Problematic internet use (PIU) has been seen as a risk factor for mental health symptoms [18, 19] and also as an important explanatory factor that facilitates the development and maintenance of SWB. Previous studies have shown the association between PIU and the SWB of adolescents [20–22]. Mei et al. (2016) pointed out that the problematic and maladaptive internet users, as compared with those adaptive users, were generally less likely to report better SWB [21]. Lin et al. (2018) also found that PIU was significantly and independently associated with lower SWB [20]. Going further, Yu & Shek (2018) indicated that poor SWB in adolescents was the consequence rather than the cause of PIU [22].

Peng et al. (2022) indicated that apart from PIU, screen time was also a risk factor for mental health [18], while Hassan et al. (2020) discovered that PIU was significantly associated with time spent daily on the internet [23]. Furthermore, Lin et al. (2011) pointed out that the average internet usage per week positively predicted PIU [24]. Anand et al. (2018) also indicated that time spent on the internet positively predicted PIU among university students [25].

While most of the abovementioned studies were conducted before the COVID-19 pandemic, recent research has shown similar patterns for PIU in the post-pandemic era. Alimoradi et al. (2022) found that the prevalence of behavioral addiction during lockdown was higher compared to non-lockdown periods, with the population of internet users being associated with overall prevalence rates for behavioral addiction [26]. Ruckwongpatr et al. (2022) demonstrated increased internet use during the COVID-19 pandemic, possibly serving as a coping strategy, while smartphone overuse may lead to PIU and its associated negative consequences [19].

### The mediating role of self-esteem

Previous research has increasingly underscored the significant role of self-esteem as a predictor of SWB [27, 28] and as a mediating factor in the relationships between various factors and SWB [29–33]. For instance, Greger et al. (2017) conducted a study involving 400 adolescents and found that self-esteem was a mediator in the relationship between childhood maltreatment and SWB [29]. Similarly, Jia et al. (2017) examined 692 migrant students across different grades and identified that self-esteem mediated the link between perceived discrimination and SWB [30]. Furthermore, Schwager et al. (2020) investigated 169 students in Germany and highlighted self-esteem’s mediating role in the relationship between social integration and SWB [28]. Additionally, Wang et al. (2017) studied 696 adolescents aged 17 to 24 and revealed that self-esteem was a mediator in the connection between social networking site usage and users’ SWB [32].

Within the framework of the Stress and Coping Model proposed by Lazarus and Folkman (1984) [34], it is evident that self-image tends to show a sense of vulnerability and threat when confronted with stressful events such as PIU. This perceived threat to self-image may significantly impact an individual’s self-esteem, which in turn is an essential factor influencing an individual’s SWB.

### The serial mediating effect of problematic internet use and self-esteem

**Fig. 1:**
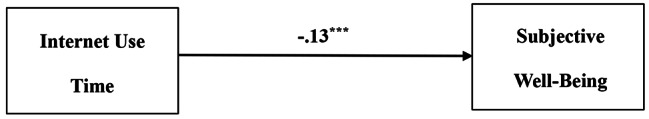
Direct effect model of Internet use time on subjective well-being. ^***^p < .001

**Fig. 2:**
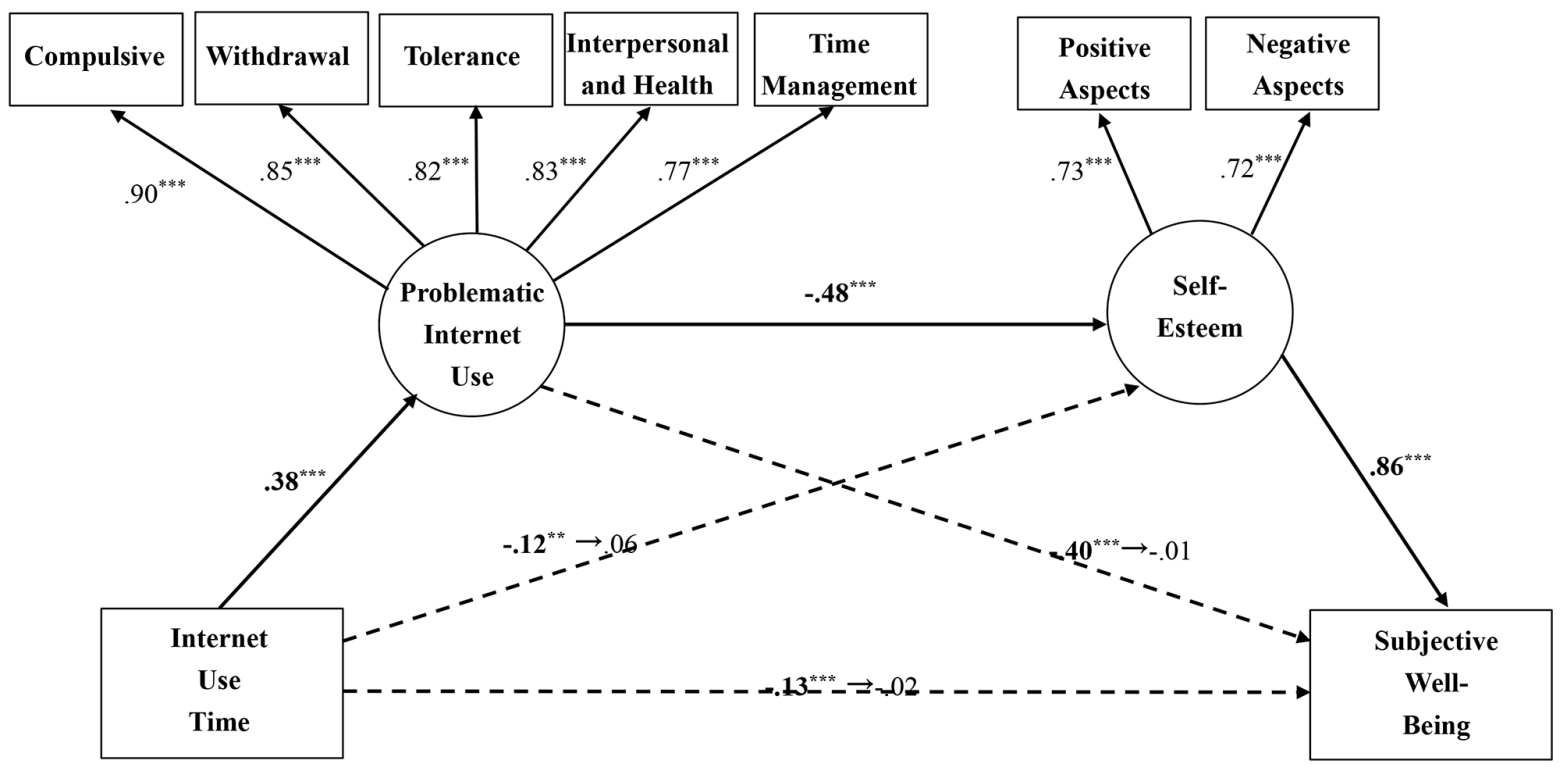
Serial mediational model. Chi-square: 225.31^***^ (df: 23); GFI: 0.95; CFI: 0.96; NFI: 0.96; NNFI: 0.94; IFI: 0.96; SRMR: 0.04; ^***^p < .001

Due to the COVID-19 outbreak, stay-at-home quarantines and mandates have stimulated the consumption of internet usage, and therefore, PIU behaviors could cause latent problems during lock-downs and become even more escalated in the teenage population [35–37]. Combining the aforementioned previous research with the Stress and Coping Model [34], we proposed that when adolescents perceive themselves as overusing the internet and experiencing PIU, it can threaten their self-image and self-concept, leading to a decline in self-esteem and, consequently, a decrease in SWB. Since no study has yet explored the mediating effect of self-esteem in the predictive relationship of PIU on SWB, the hypotheses for this study are as follows:

1. Internet use time is significantly negatively correlated with subjective well-being (Fig. 1).
2. Problematic Internet use and self-esteem sequentially mediate the negative relationship between internet use time and subjective well-being. The serial mediation model hypothesized for this study is shown in Fig. 2.

## Methods

### Participants and procedure

This study was conducted in Taiwan with a cross-sectional design during the COVID-19 outbreak. The first confirmed case of COVID-19 in Taiwan occurred on January 28, 2020, followed by an outbreak of COVID-19 in February, which resulted in a two-week postponement of the second semester. From March 2 to March 27, 2020, participants were selected from junior high schools across Northern Taiwan using stratified (Taipei City, New Taipei City, and Taoyuan City) and cluster sampling (by class). A total of 1,244 junior high school students participated in this study, which was conducted when the in-person classes were regained after the COVID-19 outbreak. Of them, 1,060 students participated (Mage = 14.66, SD = 0.86 years), resulting in a response rate of 85.21%.

The study protocol was approved by the Research Ethics Committee in National Taiwan Normal University (No. 201812HS024). The research team obtained full consent from the principals and guidance team of each school. Before administrating the survey battery, teachers of the participating classes gave consent. The research team provided administrative guidance and training to all of the counseling teachers that were responsible for the survey administration. Surveys were distributed to the students and asked to complete and return the questionnaires during class time. Participants were given full information pertaining to the purpose of the study and voluntary participation was emphasized. The research team highlighted the confidentiality of the surveys and participants signed written consent forms prior to administering the questionnaire. In addition, after obtaining participants’ consent, guardian consent was also obtained before official administration (Administrative, teachers, students, and parents all gave consent before questionnaires were given out). In order to increase participant motivation, apart from stating the purpose of the study, the process, and answering of questions when students filled out the questionnaire, counseling teachers also noted that the research team will send feedback of their data to each participant at the end of the semester. If any questions arise during or after the process of the administration, participants were free to ask for assistance.

### Measures

#### The demographic measures

Gender, age, and average hours of internet use per week were assessed.

#### Chinese internet addiction scale-revised

This scale is a 26-item self-reported questionnaire measured with a 4-point scale with scores ranging from 26 to 104 [38], and a higher total score represents the higher levels of PIU. This scale included 5 subscales, containing “Compulsive Use of Internet” (5 items), “Tolerance” (4 items), “Withdrawal” (5 items), “Time Management Problems” (5 items), and “Interpersonal and Health-Related Problems” (7 items). Of each subscale, the Cronbach’s alpha coefficients ranged from 0.78 to 0.81, and the split-half reliability over two weeks is 0.83 [38]. A Cronbach’s alpha of 0.93 was obtained when the scale was employed with Taiwanese adolescents [20], and the internal consistency coefficient for the total scale was 0.95 in this study.

#### The rosenberg self-esteem scale

The Rosenberg Self-Esteem Scale is a 10-item self-reported questionnaire to evaluate global self-esteem [39]. This scale was rated on a 6-point scale, ranging from 1 *= strongly disagree* to *6 = strongly agree*. Of the scale, 5 items describe the positive aspects and 5 items describe the negative aspects of one’s self-assessment. This study reverse-coded the negative aspects during the analysis, yielding a Cronbach’s alpha coefficient of 0.89. When administered to Taiwanese adolescents, the scale showed internal consistency coefficients of 0.91 and 0.88 for the positive and negative subscales, respectively [40]. According to confirmatory factor analysis using structural equation modeling and Rasch analysis, reliability and validity were tested for the positive and negative subscales of the Rosenberg Self-Esteem Scale, which displayed evidence that supported the reliability and validity of the Rosenberg Self-Esteem Scale [41]. Furthermore, the scale’s psychometric properties were investigated using the rating scale model (RSM), which revealed that the scale demonstrated suitability in accurately distinguishing individuals with moderate self-esteem [42].

### Chinese happiness inventory

SWB was assessed using the Chinese Happiness Inventory [43]. The SWB is a 10 item self-report questionnaire rated on a 4-point scale which examined the participants’ feelings of SWB through one of the four statements. A higher score indicated higher levels of SWB. The Chinese Happiness Inventory demonstrated good reliability and validity among adolescents in Taiwan [43]. The Cronbach’s alpha coefficient for this scale was 0.90 in the present study.

### Statistical analyses

SPSS for Windows version 18.0 was used for data analyses, and the significant level was set at *p* < .05. Pearson correlation coefficients were conducted to determine the relationship among all measures. Moreover, structural equation modeling analyses with the robust maximum likelihood method were performed by AMOS 18.0 to investigate the mediating roles of PIU and self-esteem on the relationship between internet use time and SWB. The study employed a variety of complementary fit indices to assess the model fit, including the Goodness of Fit Index (GFI), Comparative Fit Index (CFI), Incremental Fit Index (IFI), the Bentler-Bonett Normed Fit Index (NFI) and Bentler-Bonett Non-Normed Fit Index (NNFI), which consider the degrees of freedom of the model. Values greater than 0.90 were regarded to be acceptable by convention [44]. Furthermore, this study examined the serial mediation model by bootstrapping analyses [45]. This study did not incorporate data from participants with missing values for bootstrapping analyses, and therefore 34 participants were excluded from the serial mediation analysis, yielding a final sample of 1,026 participants in the structural equation modeling analyses.

## Results

### Descriptive statistics

In Table 1, the means, standard deviations, and Pearson correlations for the independent and dependent variables are presented. The average internet use time in the junior high school students sample during the COVID-19 outbreak was 24.77 h of internet usage per week (*SD* = 21.31). The correlations displayed that internet use time was significantly and positively correlated with total PIU levels and its five subscales but was negatively related to SWB and total self-esteem and its two subscales, respectively. Moreover, total PIU levels and its five subscales were significantly and negatively correlated with SWB and total self-esteem and its two subscales, respectively. Furthermore, total self-esteem and its two subscales and SWB were positively correlated with one another, respectively.

**Table 1:** The correlations among variables

Variables	1	2	3	4	5	6	7	8	9	10	11
1. Subjective well-being	―										
2. Average hours using the internet per week	− 0.13^***^	―									
3. Problematic internet use	− 0.39^***^	0.37^***^	―								
4. Compulsive use of internet	− 0.37^***^	0.32^***^	0.91^***^	―							
5. Withdrawal	− 0.35^***^	0.31^***^	0.87^***^	0.80^***^	―						
6. Tolerance	− 0.27^***^	0.33^***^	0.86^***^	0.73^***^	0.71^***^	―					
7. Interpersonal and health-related problems	− 0.36^***^	0.27^***^	0.89^***^	0.75^***^	0.66^***^	0.68^***^	―				
8. Time management problems	− 0.33^***^	0.38^***^	0.83^***^	0.66^***^	0.61^***^	0.65^***^	0.70^***^	―			
9. Self-esteem	0.72^***^	− 0.10^**^	− 0.37^***^	− 0.36^***^	− 0.31^***^	− 0.28^***^	− 0.33^***^	− 0.30^***^	―		
10. Positive aspects	0.65^***^	− 0.08^**^	− 0.25^***^	− 0.25^***^	− 0.21^***^	− 0.17^***^	− 0.23^***^	− 0.22^***^	0.85^***^	―	
11. Negative aspects	0.61^***^	− 0.08^**^	− 0.38^***^	− 0.36^***^	− 0.33^***^	− 0.31^***^	− 0.34^***^	− 0.30^***^	0.89^***^	0.52^***^	―
*M*	24.36	24.77	53.20	10.04	10.80	9.12	13.61	9.63	37.49	18.70	18.80
*SD*	6.29	21.31	16.26	3.72	3.95	3.03	4.57	3.41	10.33	5.42	6.40

^**^p < .01; ^***^p < .001

### The mediating role of PIU and self-esteem

The present study investigated the hypothesized serial mediation model as shown in Fig. 2 by using the structural equation modeling analyses. The fit indices in the serial mediation model were 0.953 on the GFI, 0.961 on the CFI, 0.957 on the NFI, 0.939 on the NNFI, 0.961 on the IFI, and 0.0389 on the SRMR. The measurement model illustrated a good fit. As a result, the entire fit of the serial mediation model was sufficient and capable of explaining 74.4% of the variance. The model presented in Fig. 1 showed that internet use time had a significant association with SWB. However, the association between internet use time and SWB decreased from − 0.13 (*p* < .001) to − 0.02 (*p* = .355) when PIU and self-esteem were included in the analysis.

On the other hand, this study investigated the indirect effect of internet use time on SWB via PIU by bootstrapping analyses with 10,000 bootstrap samples [45]. Results revealed that the mean indirect (standardized) effect of internet use time on SWB via PIU was 0.379, and bias-corrected 95% confidence interval was between 0.319 and 0.435, which did not include zero, and therefore the indirect effect was statistically significant (*p* < .001). However, the mean direct (standardized) effect of internet use time on SWB was 0.031, and the bias-corrected 95% confidence interval was between − 0.030 and 0.095, which included zero, thus the direct effect was statistically non-significant (*p* = .317). The study confirmed the full mediation model between the paths from internet use time to SWB, fully mediated by PIU.

Furthermore, this study also inspected the indirect effect of PIU on SWB via self-esteem by bootstrapping analyses. Results showed that the mean indirect (standardized) effect of PIU on SWB via self-esteem was − 0.453, and bias-corrected 95% confidence interval was between − 0.522 and − 0.381, which did not include zero, and therefore the indirect effect was statistically significant (*p* < .001). However, the mean direct (standardized) effect of PIU on SWB was − 0.018, and bias-corrected 95% confidence interval was between − 0.090 and 0.062, which included zero, thus the direct effect was statistically non-significant (*p* = .646). The full mediation model with the path from PIU to SWB was fully mediated by self-esteem.

## Discussion

Based on past literature, the study aimed to examine whether internet use time of adolescents is associated with SWB during COVID-19 outbreaks and whether PIU and self-esteem mediated the relationship between the two. In accordance with previous research, the present study discovered that self-esteem was positively correlated with SWB [27–33], and internet use time and PIU were negatively related to SWB [15, 16, 20–22, 46], respectively. More importantly, the current study found that internet use time predicted an increase of PIU, which fully mediated internet use time and SWB, and PIU predicted a decrease of self-esteem, which acted as a full mediation role between PIU and SWB among the adolescent participants during the COVID-19 outbreak.

### The relationship between problematic internet use and self-esteem

Self-esteem has been acknowledged as an essential internal resource and represents a subjective evaluation of how an individual customarily thinks and maintains about himself/herself [29, 32]. The COVID-19 outbreak came unexpectedly and has become a significant stressor for adolescents. Stay-at-home quarantines and mandates have escalated the consumption of internet usage, and therefore, PIU may cause potential problems during lockdowns, which consequently influences the teenage population at large [35–37, 47]. Furthermore, Armstrong et al. (2000) investigated the relationship between PIU and low self-esteem and indicated that problematic internet users use the internet as an escape [48]. Griffiths (2004) provided a case study perspective that described how Individuals with low self-esteem may perceive the internet as a compensative place to deal with a sense of self-deficiency [49]. Although internet usage is a daily necessity during the stressful COVID-19 outbreak, too much dependence on the internet may turn into a method of escape from reality, causing an escalation of addictive tendencies among adolescents. Compared to the normal internet usage and daily living of what life was like prior to the outbreak, life after COVID-19 may greatly affect one’s daily living styles due to overusing the internet to the extent of PIU. Not only is this drastic lifestyle change harmful to adolescents’ internal resource, but may also decrease their subjective evaluation.

### The relationship between self-esteem and subjective well-being

Examining the association between self-esteem and SWB among junior high school students during COVID-19 outbreak, structural equation modeling analyses showed that self-esteem positively predicted SWB (β = 0.86). Compared with previous studies, the current study revealed that self-esteem explained a large proportion of the variance in SWB in the well-being mediation model. Jia et al. (2017) examined 692 migrant students from fifth to eighth grade and the result showed that self-esteem mediated the relationship between perceived discrimination and SWB [30]. Schwager et al. (2020) inspected 169 students among 10 classes of three secondary schools in Germany and pointed out that self-esteem mediated the association of social integration on mental and physical well-being [28]. Additionally, Ji et al. (2019) surveyed 210 people of Chinese ethnicity with physical disability and discovered that the relationship between social support and SWB was significantly mediated by self-esteem [27]. Wang et al. (2017) tested 696 adolescents ranging in age from 17 to 24 years old and indicated that the relationship between social networking site usage and users’ SWB was mediated by self-esteem [32]. SWB reflects an individual’s cognitive and affective perceptions of life quality. Compared with previous studies, the results of the present study found that self-esteem explained a higher proportion of the SWB variance in the serial mediation model of well-being. A probable reason may be the result of forced isolation during the COVID-19 outbreak, in which adolescents were mandated to stay at home. Consequently, the lack of interaction with peers and an increased self-alone time can cause a decrease in a variety of external resources that may lead to well-being and positive self-worth, which is one of the main factors associated with SWB.

### The serial mediation model

The present study expands our knowledge by illustrating how self-esteem mediates the association between PIU and SWB. To our knowledge, this relationship has yet to be examined in any prior studies. The full mediation model was in accordance with the theoretical framework of the stress-and-coping model [34], which confirmed our second research hypothesis. The COVID-19 outbreak has significantly interrupted daily behaviors of adolescents, and repeated lockdowns and lifting of restrictions resulted in a sudden and unpredictable loss of many previous activities that was enjoyed, creating a radical change in people’s daily lives. Stay-at-home quarantines and mandates have intensified the consumption of internet usage [50], and consequently PIU could arise problems of adolescents [35–37, 47]. When adolescents consider a negative event (e.g., stay-at-home quarantines increased internet usage time, which escalated the development of PIU) as stressful, they feel their self-image threatened, which has a significant and negative prediction for adolescents’ self-esteem, and in turn is directly and fully linked with the decrease levels of SWB.

### Limitations and recommendations

The present study underlined the association between internet use behavior and SWB, and assumed to deliver a probable latent process in the relationship. However, several limitations need to be taken into consideration when interpreting the findings. First, regarding the mediating model, the present research had a cross-sectional design in nature during the COVID-19 outbreak, thus the causal relationship of internet use time, PIU, self-esteem, and SWB cannot be fully illuminated. Although self-esteem can account for a large part of the predictive associations between PIU and SWB, longitudinal studies are needed in future studies to test the meditational model. Second, despite a large sample size, generalizations from our data should be carefully assessed since the sample comprised of solely junior high school students in Taiwan. Consequently, generalizations of our findings to other countries/cultures and age groups may need further consideration. Third, Gao et al. (2020) discovered that leisure-time internet use, but not work-time internet use, was positively associated with PIU [51], and individuals who were able to balance their work-time internet use showed a higher perceived quality of life compared to participants with little amounts of internet use for work. The present study adopted an overall internet use time, and future studies can differentiate between various motives for internet use (e.g., learning time, interpersonal communication, and leisure activity). In addition, differentiation can also be made toward differing types of internet activities and PIU behaviors in order to examine the relationship between self-esteem and SWB. Lastly, all data collected in this study were self-reported, which may have inflated the relationship among variables. It would be optimal to utilize multiple methods and rating systems in future studies.

## Conclusion

The COVID-19 outbreak threatened adolescents’ mental health and livelihoods, which lowers their SWB. This study examined a serial mediational model to further analyze the relationship of internet use time, PIU, self-esteem and SWB among junior high school students by using structural equation modeling and bootstrapping analyses in a cross-sectional study design during the COVID-19 outbreak. The purpose of the present study was to determine whether the relationship between internet use time and SWB was mediated by PIU, and PIU and SWB was mediated by self-esteem, respectively. Results displayed that internet use time was able to negatively predict the levels of SWB in a large sample of junior high school students. Yet this relationship was fully mediated by PIU. In addition, PIU was also able to negatively predict the levels of SWB, which was fully mediated by self-esteem. The result is consistent with the theoretical framework of the stress-and-coping model, indicating that the fluctuation of SWB depended on how adolescents perceive their self-image to be threatened, which had a significant and negative prediction for self-esteem during the COVID-19 outbreak. Internet use time and PIU might play a distal factor role. The results from the study provide evidence in explaining how increased internet use time was associated with a greater level of PIU, which was related to lower self-esteem, and correlated with lower SWB in adolescents. The results might help mental health organizations and educational agencies to design suitable SWB promotion programs geared toward the junior high school population during a pandemic.

## Data Availability

The datasets analyzed in the current study are available from the corresponding author on reasonable request.
